# Posttranslational modifications of proteins in diseased retina

**DOI:** 10.3389/fncel.2023.1150220

**Published:** 2023-03-30

**Authors:** Christopher R. Starr, Marina S. Gorbatyuk

**Affiliations:** Department of Optometry and Vision Science, University of Alabama at Birmingham, Birmingham, AL, United States

**Keywords:** post translation modification, diabetic retinopathty, glaucoma, inherited retinal degeneration (IRD), PTMomes

## Abstract

Posttranslational modifications (PTMs) are known to constitute a key step in protein biosynthesis and in the regulation of protein functions. Recent breakthroughs in protein purification strategies and current proteome technologies make it possible to identify the proteomics of healthy and diseased retinas. Despite these advantages, the research field identifying sets of posttranslationally modified proteins (PTMomes) related to diseased retinas is significantly lagging, despite knowledge of the major retina PTMome being critical to drug development. In this review, we highlight current updates regarding the PTMomes in three retinal degenerative diseases—namely, diabetic retinopathy (DR), glaucoma, and retinitis pigmentosa (RP). A literature search reveals the necessity to expedite investigations into essential PTMomes in the diseased retina and validate their physiological roles. This knowledge would accelerate the development of treatments for retinal degenerative disorders and the prevention of blindness in affected populations.

## Introduction

**FIGURE 1:**
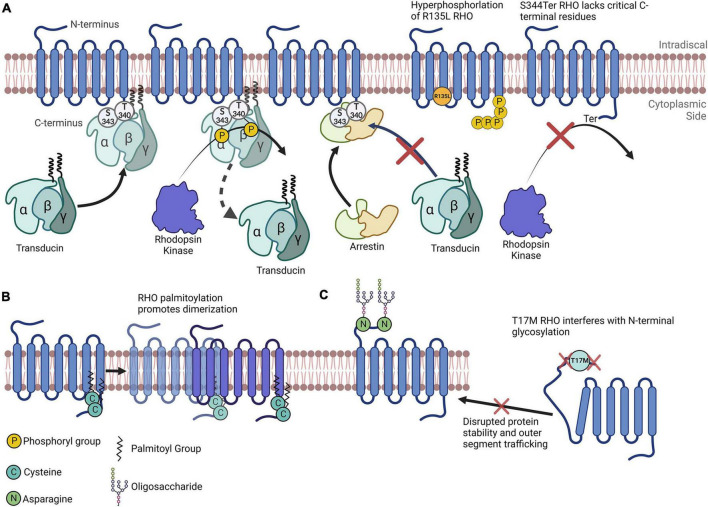
Rhodopsin as a model for understanding the importance of posttranslational modifications. **(A)** Simplified depiction of rhodopsin phosphorylation. Transducin’s interaction with RHO is impeded by RHO’s phosphorylation by rhodopsin kinase at multiple C-terminal amino acids. Arrestin preferentially binds to RHO that has been phosphorylated. The R135L RHO mutation results in RHO hyperphosphorylation. S334Ter RHO lacks many C-terminal residues and therefore lacks critical phosphorylation sites. **(B)** RHO is palmitoylated at two C terminal cysteines that promotes its homodimerization. **(C)** N-terminal N-glycosylation of RHO plays a role in its protein folding, stability and membrane trafficking. The T17M mutation interferes with the enzymatic glycosylation of the N-terminal asparagine residues, impeding RHO folding and outer segment trafficking.

Glaucoma, and diabetic retinopathy (DR) comprise a group of common eye disorders that, left untreated, often lead to blindness in 8, and 1% of cases, respectively (Taylor, 2019). Inherited retinal degeneration—including retinitis pigmentosa (RP)—is a progressive retinal disorder with a prevalence of one case in 3,000 individuals; it affects over two million people worldwide and causes severe vision loss or even blindness (Sahel et al., 2014). The molecular mechanisms underpinning retinal pathogenesis are complex and include the activation of oxidative stress, inflammation and unfolded protein responses (UPRs). Studies identify several proteins of the retina whose changing levels can have an impact on retinal disease progression, however, as most proteins are tightly controlled, the modifications of proteins have just as large (or perhaps an even larger) a role in retinal homeostasis as does protein synthesis. Protein functions are often regulated by posttranslational modifications. In this review, we highlight critical posttranslational modifications in retinal cells in healthy tissue and explore recent updates regarding PTMome in three retinal diseases: DR, glaucoma, and RP. Though there are studies on PTMs in various other retinal diseases, the scope of this review will be limited to these three diseases. Rhodopsin (RHO) is perhaps the most studied retinal protein, and its PTMs have been studied extensively—largely owing to its abundance and to its wide applicability as a classical G-protein coupled receptor. Therefore, throughout the review, we will refer to Figure 1 as we use this protein to effectively demonstrate how changes in PTMs can have drastic implications for both protein dynamics and the progression of neurodegenerative diseases.

### Phosphorylation

Phosphorylation, the most widely studied PTM and perhaps the most abundant, is the covalent linkage of a phosphate group ([PO4]3–) to an amino acid containing a free hydroxy (–OH) group (serine, threonine, or tyrosine), in a reaction catalyzed by an enzyme called a kinase (Phosphatases are the enzymes that dephosphosphorylate phosphorylated amino acids) (Table 1). Phosphorylation is most often associated with the activation or inactivation of protein typically resulting from molecular conformational changes. Although the precise mechanisms vary widely, the change in activity is often due to the phosphorylation either altering the binding partners of the protein or, in the case of autophosphorylation, resulting in an enzyme’s active site being in proximity to phosphorylatable residues. For example, the phosphorylation of RHO proteins in the retina results in its diminishing ability to stimulate transducin and phosphodiesterase activity (Figure 1A; Hurley et al., 1998).

**TABLE 1:** Summary of referenced PTM studies.

Protein	PTM	Disease/Model	References
—	*O*-GlcNAcylation	DR	Donovan et al., 2014; Xu et al., 2018
ChREBP	*O*-GlcNAcylation	DR/mice	Kim et al., 2017
IgG	N-Glycosylation	DR	Wu et al., 2021
Synaptophysin	N-Glycosylation	DR/rats	D’Cruz et al., 2012
Akt, IR, IRS-2, PDK1	Phosphorylation	DR/tree shrews and DR/mice	Kondo and Kahn, 2004; Gorbatyuk et al., 2021; Zhou Z. et al., 2022
αA-crystallin	Phosphorylation	DR and DR/mice	Ruebsam et al., 2018
STK25, GSK-3β	Phosphorylation	DR/mice	Zhou Z. et al., 2022
Src kinases	Phosphorylation	DR/mice	Sergeys et al., 2020
Rac1	Palmitoylation	DR/endothelial cells	Veluthakal et al., 2015; Alka et al., 2022
N-Ras, RhoA, calnexin, Hsp40	Palmitoylation	DR/endothelial cells	Wei et al., 2014, 2020;
Histones H3, H4, H2A, H2B, and H1	Methylation	DR/rats	Wang et al., 2017
Tau	Phosphorylation	Glaucoma/mice	Chiasseu et al., 2016
Akt	Phosphorylation	Glaucoma/mice	Edwards et al., 2020; Basavarajappa et al., 2023
Bad, S1PR1	Phosphorylation	Glaucoma/mice	Ju et al., 2018; Basavarajappa et al., 2023
GSK-3β	Phosphorylation	Glaucoma/cell line/mice	Guo et al., 2016; Fan et al., 2021
BDNF	Phosphorylation	Glaucoma/rats	Bai et al., 2010
CamkIIα	Phosphorylation	Glaucoma/mice	Guo et al., 2021
Elk-1	Phosphorylation	Glaucoma/mice	Noro et al., 2022
NfH, Tau	Phosphorylation	Glaucoma/mice	Wilson et al., 2016
Histone H3	Methylation	DR/mouse RGC line	Yu X. et al., 2022
RHO	N-linked glycosylation, phosphorylation, palmitoylation	RP/mice/cells	Wang et al., 2005; Senin et al., 2006; Murray et al., 2015
PRCD	Palmitoylation	RP/mice	Murphy and Kolandaivelu, 2016
RP2	Lipidation	RP/mice/cell line	Demers et al., 2015, Kumeta et al., 2018
NRL	Phosphorylation	RP	Kanda et al., 2007

List of PTMs highlighted in this review with their associated retinal disease and references.

### Acetylation

Acetylation is the addition of an acetyl group (donated by acetyl-coenzyme A) to a protein at either the N-terminal α-amino group or the amino side chain of lysine residues [reviewed extensively in Drazic et al. (2016) and Narita et al. (2019)]. The enzymes involved in this process are N-terminal acetyltransferases and lysine acetyltransferases. Acetylation can have a profound impact on a protein’s role in the cell, by altering attributes such as protein localization and stability. Histone acetylation is the classical process that has a significant impact on gene expression. Non-histone acetylation is less studied but there is evidence suggesting that this PTM shapes cellular dynamics through the acetylation of nonhistone proteins. Deacetylation is carried out by deacetylases.

### Methylation

Methylation is the addition of a methyl (–CH3) group to an amino acid with a nitrogen-containing side chain, most frequently lysine and arginine. The methylation of histones has been the focus of most studies examining protein methylation, as it alters chromatin structure and therefore gene expression; nonhistone methylation, meanwhile, changes various cellular processes, including signaling, DNA repair, transcription, and translation [reviewed in Gong and Miller (2019) and Di Blasi et al. (2021)].

### Lipidation

Protein lipidation controls many physiological aspects. The effects of protein lipidation on cellular function include the regulation of protein–membrane and protein–protein interactions, functional stability, and enzymatic activities. The major lipid modifications of proteins, occurring near the C-terminus of proteins, include prenylation, myristoylation, and palmitoylation (Jiang et al., 2018). Prenylation is the addition of multiple isoprene moieties to cysteine residues near the C-termini of proteins. This phenomenon is observed in about 2% of all cellular proteins in mammalian cells. Given that, two types of prenylation—namely, farnesylation and geranylgeranylation—are known to take place in mammalian cells. The linkage between the farnesyl or geranylgeranyl groups and the cysteine residues of proteins is a thioether bond, the formation of which is believed to create an irreversible PTM that has no known enzyme that can reverse this modification in intact proteins. Farnesyl transferase transferring the 15-carbon farnesyl group from farnesyl diphosphate to substrate proteins, and geranylgeranyl transferase catalyzing a similar reaction comprising the transfer of a 20-carbon geranylgeranyl group from geranylgeranyl diphosphate, are members of the protein prenylatranferase family (Jiang et al., 2018). Examples of protein lipidation include farnesylation of the retinal RHO kinase, G protein-coupled receptor kinase (GRK) 1, and geranylgeranylation of GRK7 in the retina (Zhang et al., 2004; Wada et al., 2006). Lipidation of GRK1 and GRK7 appears to be essential for their incorporation into lipids and its transport to the outer segment, both of which subsequently reduces their enzymatic activity (Wada et al., 2006; Zhang et al., 2007; Millette et al., 2022).

*Myristoylation* involves the posttranslational attachment of a saturated 14-carbon fatty acyl group, myristoyl, to the N-terminal glycine of proteins. This process is controlled by N-myristoyltranfetase 1 and 3 (NMT1 and NMT2). In humans, NMT1 and NMT2 share approximately 76% sequence identity and have partially overlapping biological functions and substrate selectivity. A study conducted with retinal pigment epithelial (RPE) cells infected with herpes simplex virus demonstrated a high level of myristoylation of ADP-ribosylation factor (ARF) proteins 1, 3, 4, 5, and 6 that play an important role in membrane trafficking, actin remodeling, and secretory and endocytic pathways (Serwa et al., 2015).

*Cysteine palmitoylation* (or S-palmitoylation) refers to the addition of a 16-carbon palmitoyl group *via* thioester bonds on protein cysteine residues. This reaction is highly reversible and dependent on enzymatic or nonenzymatic hydrolysis (Jiang et al., 2018). Compared to other types of lipidation, S-palmitoylation lacks a specific sequence motif; this makes it is difficult to predict a candidate undergoing the reaction. Nevertheless, a major pattern of S-palmitoylation exists in cells, in that it typically occurs on cysteines near or within a transmembrane domain or near a membrane-targeting PTM, such as prenylated cysteine or N-terminal myristoylated glycine (Jiang et al., 2018). Palmitoyl transferases belong to the class of Golgi apparatus-specific proteins containing the aspartic acid–histidine–histidine–cysteine (DHHC) cysteine-rich zing finger domain (Jiang et al., 2018). Recent studies demonstrated the presence of palmitoyltransferases zinc finger DHHC-type palmitoyltransferase 17 (ZDHHC17) and Serine palmitoyltransferase, long chain base subunit 1 (SPTLC1) in the retina (Gantner et al., 2019; Niu et al., 2020). Examples of RPE and photoreceptor (PR)-specific palmitoylated proteins include RPE65 and RHO (Figure 1B; Seno and Hayashi, 2017; Uppal et al., 2019).

*Glycosylation* is the known process during which sugar moieties attach to proteins. The main types of glycosylation are *N*-linked glycosylation, *O*-glycosylation, glycosphingolipidation, and glycosaminoglycation (Reily et al., 2019). *N*-glycans play a crucial role in regulating a variety of intracellular and extracellular functions. *N*-linked glycosylation refers to the attachment of *N*-acetylglucosamine (GlcNAc) to a nitrogen atom of asparagine amino acids. These conjugates contain a GlcNAc mannose core, the number of which is modified during the process of protein glycosylation to form the 14-sugar structure Glc_3_Man_9_GlcNAc_2_. To form this structure, *N*-glycosylation requires the formation of a lipid precursor, a branched carbohydrate structure of GlcNAc and Man attached to dolichol phosphate on the cytoplasmic side of the endoplasmic reticulum (ER). This lipid precursor is then flipped into ER lumen and Man, and glucose units are added to form the 14-sugar structure Glc_3_Man_9_GlcNAc_2_. The stability of RHO and its incorporation into the PR outer segment disc strongly depend on its glycosylation status (Murray et al., 2015).

*O*-*glycosylation* occurs on Ser and Thr amino acids. The most common sugar moieties linked to these amino acids are GlcNAc and N-acetylgalactosamine (GalNAc). GlcNAc linked to Ser and Thr often presents on intracellular glycoproteins in the nuclear, mitochondria, and the cytoplasm. The addition of GlcNAc transfer is regulated by *O*-GlcNAc transferase (OGT) and *O*-GlcNAcases. These enzymes are known to provide a rapid cycle of adding and removing GlcNAc from proteins, although additional PTM (protein phosphorylation at Ser and Thr residues) competes with O-GlcNAc (Schjoldager et al., 2020).

### Glycation

Protein glycation is a PTM involving a chemical reaction between primary amine groups of proteins and an aldehyde or hemiacetal group of aldose sugars such as D-glucose, leading to the generation of advanced glycation end product (AGE) adducts (Rabbani and Thornalley, 2008). The formation of AGEs occurs on proteins with a slow turnover rate. It occurs predominantly on lysine, arginine, and N-terminal residues of proteins and usually occupies up to 5% of protein sites. Protein glycation is highly dependent on the local environment of the primary amine groups present on the surface of proteins. For example, for glycation, surface-exposed Lys residues require the participation of neighboring groups. Glycation may affect the protein structure and functions and cause immunogenicity. AGEs are also known to be toxic to humans. The receptor of AGEs—known as RAGEs—also demonstrates an affinity to amyloids. The glycation of serum albumin, hemoglobin, and serum immunoglobulins has been extensively studied. For example, proteins that are A1C or HbA1C is a glycated form of hemoglobin measurement, and it is the most widely accepted clinical laboratory test by which to monitor diabetes. Protein glycation is implicated in many age-related chronic diseases such as Alzheimer’s, Parkinson’s, and cardiovascular diseases, peripheral neuropathy, cancer, and diabetes.

*Glycosphingolipidation* (GSL) is a process involving the attachment of glycans to sphingolipids at the C1 hydroxyl position of a ceramide. It starts with the addition of glucose to the lipid moiety at the cytoplasmic side of the ER. The structure is then flipped into the lumen. The enzymes initiating GSL glycosylation are specific for lipids; further processing of the carbohydrate chain is carried out by glycosyltransferases. A vital role of glycosphingolipid in DR affected by AGEs in retinal microvascular cells has been elucidated (Natalizio et al., 2001). For the purposes of this review, other PTMs including ubiquitination, citrullination, and the like will not be discussed.

## Posttranslational modifications in the retina

Recent developments in proteomic research have made it possible to identify a set of proteins differentially posttranslationally modified in diseased retinas (Garcia-Quintanilla et al., 2022; Lam et al., 2022; Lee et al., 2022; Nattinen et al., 2022; Smyczynska et al., 2022; Zhou H. et al., 2022). However, despite advancements in molecular knowledge regarding the retinal pathobiology of ocular diseases, the question of whether the differential expression of proteins associates with altered activity remains unaddressed. To answer this question, researchers should conduct detailed research on PTMomes in tandem with proteomic studies in affected ocular tissues. We as researchers are still learning about major PTMs occurring in the proteins of various retinal cell types. In this review, we highlight major protein PTMs associated with the retinal tissues of animals mimicking glaucoma, DR, and inherited RP in patients.

## Diabetic retinopathy

As a life-limiting and life-threatening complication of diabetes, DR, a diabetes-associated neuromicrovascular dysfunction, currently affects 100 million people worldwide (Duh et al., 2017). Type-1 diabetes (T1D) and type-2 diabetes (T2D) differ, in that T1D—also known as juvenile diabetes—is an autoimmune metabolic disorder wherein the pancreas produces little to no insulin, while patients with T2D create insulin but their body develops resistance to its use, resulting in an excess of glucose in the blood. This excess can be injurious to numerous body organs and tissues. Typically, T1D requires insulin injection, appears earlier in youth, and demonstrates less prevalence than T2D. DR falls into two major categories: early, nonproliferative DR (NPDR) and advanced proliferative DR (PDR). Patients with NPDR manifest visible features, including microaneurysms, retinal hemorrhages, and intraretinal microvascular abnormalities, while patients with PDR develop pathologic preretinal neovascularization.

Overall, two major cellular events, neurodegeneration and retinal vasculature dysfunction, are known pathophysiological biomarkers of DR. Currently, pathological neovascularization is a primary focus of DR diagnosis and treatment (Juen and Kieselbach, 1990; Di Leo et al., 1994; Barber et al., 1998; Tyrberg et al., 2011; Murakami and Yoshimura, 2013; Soliman et al., 2016). However, research on retinal dysfunction—including reduced cone sensitivity (Weiner et al., 1997), the delayed activation of the cone phototransduction cascade (McAnany and Park, 2019), the selective loss of S cones (Cho et al., 2000; Nomura et al., 2000), glial abnormalities, and the thinning of both the nerve fiber layer and the retinal ganglion cell (RGC) layer—in patients with DR (Verma et al., 2009; Vujosevic and Midena, 2013; Verbraak, 2014) has been delayed due to the lack of or inaccessibility to an appropriate animal model. This delay is most likely responsible for delays in determining an active PTMome in various diabetic retinal cells.

Many systemic features of diabetes impact the retina. These features include hyperglycemia and high blood glucose level, the accumulation of AGE adducts, dyslipidemia, and hypertension; hyperglycemia accounts for 10% of all risk factors for developing DR (Hirsch and Brownlee, 2010). While the molecular mechanism of DR is complex, it is believed that aberrant glucose flux and hyperglycemia are responsible for the activation of protein kinase C, hexose monophosphate, AGE, and polyol pathways, which eventually leads to chronic UPR activation (Pitale and Gorbatyuk, 2022).

## Posttranslational modifications in diabetic retinopathy

### Glycosylation

An alteration in the protein glycosylation pattern is perhaps one of the PTMs most relevant to diabetes. The *O*-GlcNAc and N-glycosylation modifications are glucose sensors linking the pathogenesis of diabetes and hyperglycemia to various kinds of cellular signaling in the diabetic retina. Previous studies report that altered *O*-GlcNAcylation is a complication of insulin resistance that leads to diabetic pathologies such as DR (Donovan et al., 2014; Xu et al., 2018). Recent studies of the molecular mechanisms of DR reveal the contribution of altered *O*-GlcNAc to an increase in vascular endothelial growth factor expression (Donovan et al., 2014), the development of retinal neovascularization (Gurel et al., 2013), the formation of reactive oxygen species (ROS) (Liu et al., 2015), and the breakdown of the blood–retina barrier (Xu et al., 2014) in the diabetic retina.

Research conducted with vitreous samples collected from patients with PDR demonstrated that the level of OGT enzyme transferring *O*-GlcNAc to substrates is significantly elevated, suggesting increased *O*-GlcNAc levels (Donovan et al., 2014; Xu et al., 2018). Those researchers also demonstrated an increased level of O-GlcNAcylation rate in diabetic retinas. Moreover, the treatment of bovine retinal vascular endothelial cells (BRVEC) with AGE for 24 h was found to significantly increase the *O*-GlcNAcylation level. Mimicking PDR, the researchers also found that hypoxia favors increases in both *O*-GlcNAcylation pattern and OGT expression. These data were confirmed with BRVECs cluttered under high-glucose conditions.

Another study emphasized an increase in OGT in the diabetic retina (Kim et al., 2017). Thus, in diabetic retinas and RPE cells exposed to high glucose levels, the researchers identified elevated levels of carbohydrate-responsive element-binding protein (ChREBP) and thioredoxin-interacting protein (TXNIP) and the activation of nuclear factor kappa B (NF-κB) and poly (ADP-ribose) polymerase. Moreover, the *O*-GlcNAcylation of ChREBP was enhanced by the OGT enzyme. They next conducted double immunofluorescence analysis and found an increase in the colocalization of terminal deoxynucleotide transferase-mediated dUTP nick-end labelling-positive ganglion cells and OGT, ChREBP, TXNIP, or NF-κB in diabetic retinas, compared to control retinas. Therefore, the authors of that study conclude that OGT inhibition might be a neuroprotective strategy by which to delay retinal cell death (Kim et al., 2017).

The link between the N-GlcNAcylation of immunoglobulin G (IgG) and DR was recently studied in patients (Wu et al., 2021); the authors of that study conclude that certain patterns of IgG glycosylation are associated with DR. In particular, GP15, GP20, and IGP54 showed a negative trend in N-GlcNAcylation, while IGP32 demonstrated positive changes, compared to a control. Therefore, the authors propose that “the significant glycosylation panel, reflecting an aging and proinflammatory status, may capture a specific biological aspect and become a novel biomarker and drug target of DR” (Wu et al., 2021). Another study, of streptozotocin (STZ)-induced diabetic rats, demonstrated that hyperglycemia enriches the mannose rich N-glycosylation of synaptophysin (D’Cruz et al., 2012). Synaptophysin, an abundant transmembrane protein in the presynaptic neurotransmitter vesicles of neurons, is a glycoprotein with four transmembrane domains, and both amino and carboxyl termini face the cytoplasm. Under normal physiological conditions, this protein has been proposed to interact with other synaptic proteins—including the v-SNARE vesicle-associated membrane protein 2—to play a role in vesicle docking and neurotransmitter release. Under diabetic conditions and a diabetes-induced irregularity in the posttranslational processing of synaptophysin, the PTM could explain its accelerated degradation, leading to a significant decrease in the thickness of the entire retina and the inner and outer nuclear layers overall.

### Advanced glycation end product adducts

The accumulation of circulating AGEs in retinal cells strongly correlates with DR (Kang et al., 2022). Diet is a major source of AGEs, and long-term AGE intake leads to their cumulation in body fluids, contributing to the pathogenesis of diabetic retina. Under hyperglycemic conditions, AGE accumulation has been observed in vascular cells, neurons, and glia, and they may have pathogenic implications in individual cells and in retinal function (Hammes et al., 1994; Cooper et al., 2001; Gardiner et al., 2003; Sharma et al., 2012). In addition, AGE accumulation has been found in pericytes during diabetes (Stitt et al., 1997). These adducts may be responsible for pericyte loss and other characteristic changes, including thickening of the basement membrane and hyperpermeability. It has been also demonstrated that the AGE–RAGE interaction elicits ROS generation in cultured retinal pericytes, inducing the apoptotic cell death of pericytes, thus inducing NF-κB activation and reducing the B-cell lymphoma 2 (Bcl-2)/Bcl-2-like protein 4(Bax) ratio in affected pericytes (Huttunen et al., 2002; Schiekofer et al., 2003). AGEs can alter the properties of the large matrix proteins collagen, vitronectin, and laminin, through AGE–AGE intermolecular covalent bonds or through cross-linking (Schmidt et al., 1995; Hammes et al., 1996; Howard et al., 1996). Intracellularly, basic fibroblast growth factor is one of the proteins that may be glycated (Giardino et al., 1994). Overall, the adverse extracellular effects of AGE adducts include the formation of cross-links between key molecules in the basement membrane of the extracellular matrix, permanently altering cellular structure and the interaction of AGEs with RAGE on cell surfaces, thus altering cellular function. The intracellular effects of AGE include the initiation of oxidative stress, and the upregulation of NF-kB and its downstream targets (such as cytokines and endothelin-1) (Goldin et al., 2006).

### Phosphorylation

Protein phosphorylation plays a critical role in relaying the insulin signal from initiation at the insulin receptor (IR) to the transport of glucose transport protein (GLUT) type 1 and 4 to the plasma membrane. Dysregulated protein phosphorylation events in insulin signaling may contribute to various diseases, such as T2D. Extensive research has been carried out vis-à-vis the role of kinases in insulin action. For example, in STZ-induced diabetic mice with T1D, IR, IR substrate (IRS)-2 protein, and tyrosine phosphorylation were found to increase with insulin in the diabetic retina, while IRS-1 protein and its phosphorylation were maintained (Kondo and Kahn, 2004).

In these mice, Phosphatidylinositol 3,4,5-trisphosphate generation by acute insulin stimulation was enhanced in retinal endothelial cells. Meanwhile, protein levels and the phosphorylation of phosphoinositide-dependent protein kinase 1 (PDK1) and protein kinase B (AKT; S473) were decreased in the diabetic retina, with the conclusion being that in the diabetic retina, there are alterations in insulin signaling—such as impaired PDK/AKT responses—that may contribute to retinopathy. Controversially, our study of diabetic tree shrews with cone-reached retinas demonstrated an increase in p-AKT (S473) that is most likely due to a differential AKT phosphorylation pattern occurring in rod and cone PRs (Gorbatyuk et al., 2021).

A recent study demonstrated the neuroprotective role of αA-crystallin phosphorylation during diabetes. In particular, regulation by its phosphorylation on threonine (human) and serine (rodents) residue 148 was found to play an essential role in neuronal survival (Ruebsam et al., 2018). Predominantly, expressed in RGC and Müller cells, the reduction in Thr^148^-phosphorylated level was dramatically reduced in diabetic donors, especially those with DR. The neuroprotective mechanism is proposed to be associated with its chaperoning activity and the UPR associated with metabolic stress. Another study of diabetic RGC demonstrated the neuroprotective role of serine/threonine protein kinase 25 (STK25) in a mouse model of high-glucose elicited damage. The silencing STK25 strengthens the activation of the nuclear receptor Nrf2 pathway and restores the phosphorylation of AKT and glycogen synthase kinase-3β (GSK-3β) in high-glucose-challenged RGC, prompting researchers to propose an AKT-Gsk3β-Nrf2 axis as a new neuroprotective signaling for diabetic RGC (Zhou Z. et al., 2022).

The retinal tyrosine kinome of diabetic Akimba mice has recently highlighted a potential neuroprotective strategy that inhibits a specific Src family kinase known to promote vascular dysfunction (Sergeys et al., 2020). Applying human umbilical vein endothelial cell tube formation and murine organotypic choroidal sprouting assays, the authors demonstrated the shift in the Akimba retinal tyrosine kinome towards a hyperactive state and the central role of Src- focal adhesion kinase (FAK) family kinases. In that study, Src-FAK kinases inhibitors were able to suppress angiogenesis, thus providing the first retinal tyrosine kinome changes in the Akimba model of DR; the authors thus propose an attractive therapeutic intervention for retinal vascular pathology.

### Palmitoylation

This PTM has not been extensively studied in the retinal research field. Although highly dynamic and therefore challenging to detect, the palmitoylation pattern of 192 proteins including 55 novel candidates has been identified in normal human RPE cells (Li et al., 2022). Among them are immunoglobulin heavy chain proteins, integrin subunits, transcriptional factor Slit homolog 3 (SLIT3) responsible for the retinal neovascularization, and oxytocin receptor, whose role in DR has been recently highlighted (Degirmenci et al., 2019). Moreover, the authors demonstrated that these candidates form functional groups of proteins responsible for translation, cell-matrix adhesion, chaperone-containing complex, and cell surface interaction. Indeed, in the diabetic retina, free circulating palmitic acid may result in enhanced protein palmitoylation. Thus, it has been proposed that the palmitoylation of Ras-related C3 botulinum toxin substrate 1 (Rac1) at the cysteine-178 residue is responsible for the T-lymphoma invasion and metastasis-inducing protein 1(Tiam1) - Rac1- NADPH oxidase 2 (Nox2) axis-mediated activation of p38 kinase in endothelial cells treated with high glucose (Veluthakal et al., 2015). Moreover, another research group demonstrated that the inhibition of the rate-limiting enzyme and serine-palmitoyl transferase prevents increased Rac1 transcription by regulating the activity of DNA methylation-hydroxymethylation machinery, resulting in diminishing accelerated capillary cell loss (Alka et al., 2022).

A recent study conducted with endothelial cells mimicking hyperglycemic environment proposes excessive protein palmitoylation in diabetic retina and found that insulin-regulated palmitoylation impacts endothelial cells (Wei et al., 2014, 2020). Among all palmitoylated candidates, the authors identified G protein-associated candidates such as Neuroblastoma RAS viral oncogene homolog (N-Ras) and Ras homolog family member A (RhoA), transporter proteins (cation-dependent mannose-6-phopshate receptor), chaperones (calnexin, Heat shock protein 40), and others (Wei et al., 2014).

### Methylation

Overall, DNA methylation plays an essential role in both the normal and pathological development of the human retina. For example, the gene expression patterns of PR and non-PR cells in the developing retina are strongly controlled by methylation (Merbs et al., 2012). On other hand, a link was found between aberrant DNA methylation and retinal diseases, including DR (Lu et al., 2021). The role of protein posttranslational methylation in the diabetic retina has not been extensively studied; thus, in the diabetic retina, posttranslational protein methylation has been primarily investigated in histones. Wang et al. identified 266 differentially modified histone peptides, including 48 of 83 methylation marks with significantly different levels of abundance in the retinas of diabetic rats as compared to nondiabetic controls (Wang et al., 2017). That study pinpointed 155 of 266 identified peptides carrying between one and four PTMs. A total of 135 distinct histone marks were distributed as follows: 43 on histone H3, 19 on H4, 37 on H2A, 22 on H2B, and 14 on H1. The study validated results obtained with liquid chromatography–mass spectrometry and revealed the upregulated mono- and di-methylation states of histone H4 lysine 20 (H4K20me1/me2) that are markers related to the DNA damage response. Moreover, the authors found that the treatment of Müller cells cultured in high glucose with minocycline reduced the DNA-damage markers phosphorylated- ataxia telangiectasia and Rad3-related protein (ATR), phosphorylated- Breast cancer type 1 susceptibility protein (BRCA1), phosphorylated- Checkpoint kinase 1 (Chk1), and phosphorylated-p53. Altogether, this study found that the “alteration of some histone methylation levels is associated with the development of diabetic retinopathy in rodents, and the beneficial effect of minocycline on the retinas of diabetic rodents is partially through its ability to normalize the altered histone methylation levels.”

### Acetylation

Another example of PTMome that has not been broadly studied is acetylation; like histone methylation, it is a primarily studied PTM in the diabetic retina. Histone acetylation significantly increases in the retinas of diabetic rats (Kadiyala et al., 2012). Similar to the methylation of histone, histone acetylation is inhibited in diabetics treated with minocycline, a drug known to inhibit early DR in animals. Overall, an increase in histone acetylation may prompt the expression of inflammatory proteins that have been implicated in the pathogenesis of DR. The authors found that both the acetylation and induction of the inflammatory proteins in elevated glucose levels are significantly inhibited by inhibitors of histone acetyltransferase (garcinol and antisense against the histone acetylase, p300) or activators of histone deacetylase (theophylline and resveratrol), and were increased by the histone deacetylase inhibitor known as suberoylanilide hydroxamic acid. Although the authors conclude that hyperglycemia causes the acetylation of retinal histones and probably other proteins, they do not show the acetylation pattern of individual proteins in the diabetic retina.

## Glaucoma

Glaucoma, a disease caused by damage to the optic nerve, is a leading cause of blindness worldwide. The optic nerve carries information from the eye to the brain through the axons of retinal ganglion cells (RGCs). Damage to these axons results in the disruption of visual input to the brain and the progressive degeneration of RGC axons and ultimately their somas. While the pharmacological or surgical reduction of intraocular pressure (IOP) is an approved treatment for glaucoma, very little is currently understood about this disease’s pathogenesis. Therefore, it is critical to identify targets by which to promote reinnervation and neuroprotection. Jacobi et al. (2022) report that transcriptional programs for treatments that successfully promote RGC axon regeneration and cellular survival have significant overlap; however, other research groups continue to report that treatments promoting survival also simultaneously inhibit or significantly limit axon regeneration and vice versa. For example, Lindborg et al. studied 400 proteins, most of them individually, and in their best results report only modest axon regeneration and neuroprotection (Lindborg et al., 2021). The referenced reports provide evidence of a dissociation between gene or protein expression and function in injured mammalian RGCs, further supporting a critical need to understand proteins at the regulation level.

## Posttranslational modifications in glaucoma, and models of retinal ganglion cell injury

By assessing proteomic changes in human glaucomatous retina samples, Funke et al. demonstrated that the levels of a plethora of proteins are altered in glaucoma (Funke et al., 2016). Further, while studies have been published on large-scale proteomics studies pertaining to protein expression at the onset of central nervous system (CNS) neuron injury (Tonner et al., 2022), the PTMome of RGCs in neurodegenerative diseases is largely unknown. Liu et al. and Lukas et al. each assessed phosphoproteomic changes in mouse total retina lysate following optic nerve injury (Lukas et al., 2009; Liu et al., 2020). Their respective datasets appear to be the only ones to analyze PTMs on a large scale in animal models of optic nerve injury; however, because those studies assessed only phosphorylation in total retina lysate, they still failed to create a clear picture. The remaining studies that will be referenced assess PTMs on a protein-by-protein basis in optic neuropathies. In this section, we review studies that explore changes in posttranslational modifications in glaucoma.

As mentioned, most researchers have assessed the PTMs of individual proteins in diseases of RGCs. For example, Chiasseu et al. highlight the altered localization and phosphorylation of tau primarily in the dendrites of RGCs in mice in response to elevated IOP (Chiasseu et al., 2016). Furthermore, it is becoming increasingly more common to find proteins with a reduced PTM that plays a role in the progression of RGC degeneration in animal models. For example, the study of Edwards et al. is just one to report of a reduction of AKT phosphorylation at two sites, Thr308 and Ser473 (Edwards et al., 2020). Importantly, these phosphorylation sites are both vital to the enzymatic function of AKT and closely correlate with neuronal survival (Diem et al., 2001; Nakazawa et al., 2003; Tsai et al., 2010). Basavarajappa et al. report that the pharmacological activation of Sphingosine-1-phosphate receptor 1 (S1PR1) promotes neuroprotection through AKT activation and the subsequent inhibition of apoptosis through the phosphorylation of Bcl-2-associated death promoter (Bad) at Ser136 (Basavarajappa et al., 2023). The phosphorylation of Bad at Ser112 may also be important to preventing RGC cell death, as it is regularly abundant in healthy RGCs; meanwhile, Ju et al. report near-normal levels of p-Ser112 and the preservation of RGCs in a retinal ischemia model after ubiquinol treatment (Ju et al., 2018). These reports indicate that Bad regulation is likely critical to the progression of apoptosis in many diseases that impact RGCs. Similarly, multiple research groups report on the significance of GSK-3β regulation in the progression of RGC axonal degeneration (Guo et al., 2016; Fan et al., 2021). Guo et al. report that the AKT-mediated inactivation of GSK-3β by phosphorylation at Ser9 is central to AKT-mediated CNS axon regeneration (Guo et al., 2016). Interestingly, the effect of GSK-3β on axon regeneration was found to have no impact on RGC cell survival, adding to the growing number of reports on the dissociation of axon regeneration and neuroprotection (Guo et al., 2016; Lindborg et al., 2021). Brain-derived neurotrophic factor (BDNF) is another factor that has been extensively studied in animal models featuring RGC injury: Bai et al., for example, report that BDNF activation of its target receptor leads to the neuroprotection of RGCs and involves the tyrosine phosphorylation of Tropomyosin receptor kinase B (TrkB) (Bai et al., 2010).

Multiple studies identify mutant proteins that have an improved capacity to promote neuroprotection or axon regeneration, relative to their wildtype counterpart. In mouse models of RGC damage, we previously reported that the adeno-associated virus-mediated overexpression of a phosphomimetic constitutively active mutant of Calcium–calmodulin (CaM)-dependent protein kinase II αlpha (CamkIIα) (T286D) drastically elevated RGC neuroprotection compared to wildtype CamkIIα (Guo et al., 2021), directly demonstrating that an understanding of the changes in PTMs in response to injury could be critical to maximizing neuroprotective efforts. Further, Noro et al. demonstrated that RGC axonal regeneration promoted by reported ETS Like-1 (Elk-1) was further elevated by manipulating its phosphorylation sites (Noro et al., 2022). Together, these two reports directly suggest that phosphorylation can be significant in two major avenues to combat neurodegeneration, and promote axonal regeneration and neuroprotection. In addition, a constitutively active mutant AKT dramatically promotes axon regeneration *in vivo*. Likewise, it was found that overexpressing a constitutively active form of NF-κB significantly improves neuroprotection in postischemic injury RGCs. Wilson et al. (2016) report that the hyperphosphorylation of neurofilament heavy chain and tau in retinal projections preceded the same changes in the retina. As these components are important to cytoskeletal integrity and axon transport, just how the observed pattern precisely follows Wallerian degeneration is intriguing. Bhattacharya et al. (2006) report an elevation in protein deiminase 2 (Pad2) in human glaucomatous optic nerves. Pad2 is an enzyme responsible for protein citrullination. Further, the group identified several proteins with elevated citrullination, compared to control donor nerves. Another research group report that the use of valproic acid, a histone deacetylase (HDAC) inhibitor, significantly reduced RGC cell death in a rat model of ocular hypertension (Alsarraf et al., 2014). Li et al. (2018) assessed Abca1 ubiquitination in a mouse model of glaucoma and found that this PTM could be critical to regulating Annexin A1 secretion.

Multiple research groups highlight the potential impacts of histone alterations in RGCs and whether these changes could impact the progression of RGC pathology. For example, Yu et al. examined the role of an elevated lysine-specific demethylase 1 (LSD1) in oxygen–glucose deprivation/reoxygenation-induced RGC damage (Yu X. et al., 2022). Mechanistically, the group provides striking evidence of the involvement of LSD1 in RGC cell death, through its demethylation of Histone H3K4me2 and subsequent inhibition of mIR-21–5p. Their conclusion aligns with that of a 2012 study by Zhang et al. (2012) who report the RGC neuroprotective effects of broad-spectrum HDAC inhibitors following optic nerve crush. Pan et al. (2022) mapped a mutation in the gene encoding Methyltransferase-like 23 (*METTL23*) in a familial case of normal tension glaucoma. That research group demonstrated the possible significance of this mutation by demonstrating that mice overexpressing or lacking in *Mettl23* develop glaucoma-like phenotypes in the absence of elevated IOP. Yu at el. report that the acetylation of p53 may lead to a reduction in Bax/Bcl-2 interaction, and ultimately apoptosis, in RGCs (Yu H. et al., 2022).

Although it provides only a snapshot of the studies that assess altered PTMs in RGCs in diseased states, this summary hopefully serves to demonstrate the significance of protein regulation in the progression of RGC degeneration, and underscore why is it important to identify the retinal PTMome to develop novel therapeutic targets for the treatment of RGC diseases.

## Retinitis pigmentosa

Retinitis pigmentosa is a rare inherited disorder resulting in the progressive degeneration of retinal PR cells, leading to night blindness and worsening peripheral vision. Eventually, RP progresses to incurable (and legal) blindness. RP can be inherited in an autosomal dominant (ADRP), autosomal recessive, or X-linked fashion. Many forms of RP derive from a mutation in PR-specific proteins. Some of the most common mutations seen in these diseases are in the *RHO* gene—for example, P23H. RHO is a highly conserved enzyme in which many singular amino acid substitutions can have profound effects. Mutations in the genes encoding many RHO effectors can also lead to RP.

## Posttranslational modifications and retinitis pigmentosa

This review section focuses on the major PTMs identified in RP, and emphasizes PR-specific proteins. Upon exposure to light, RHO is phosphorylated at many serine and threonine residues near its carboxyl-terminus (C-terminus, Figure 1A); this can have profound effects on its function and therefore have important implications vis-à-vis the visual response (Lokappa et al., 2019). Because of this, RHO phosphorylation is tightly controlled (Hurley et al., 1998; Hsieh et al., 2022; Vishnivetskiy et al., 2022). For example, phosphorylation is critical for its recognition by Arrestin1, a RHO mediator that kickstarts the RHO inactivation process (Figure 1A; Vishnivetskiy et al., 2022). It is important to note here that this interaction is critical, as it results in inactivated RHO being largely ignored by Arrestin1. Interestingly, cone arrestin (Arrestin4) does little to inactivate RHO (Hsieh et al., 2022), further demonstrating how specific PTMs can directly drive the activities of multiple proteins. Jatana et al. (2020) show that the phosphorylation of RHO leads to conformational changes that likely drive both its change in activity and its recognition, by inactivating enzymes. RHO phosphorylation also regulates its affinity to transducin, therefore implicating this PTM in the modulation of several aspects of RHO regulation (Gibson et al., 2000). Interestingly, RHO’s phosphorylation state may dynamically alter the competitive binding of transducin and arrestin to RHO (Pulvermüller et al., 2000).

Another important note is that many single amino acid substitutions in RHO can cause RP. Some of these amino acids are modified posttranslationally, or their mutations are implicated in preventing modifications on nearby amino acids. For instance, T17M RHO, one of many RHO mutations leading to ADRP, interferes with N-linked glycosylation at Asn15 (Murray et al., 2015). In this case, this site’s inability to be glycosylated likely results in improper tertiary structure formation and the subsequent accumulation of RHO in the ER (Figure 1C; Murray et al., 2015). Another RP-causing mutation, R135L RHO, exhibits a different mechanism: R135L results in the hyperphosphorylation of RHO, which may be due to aberrant phosphorylation inhibition mediated by Ca^2+^/recoverin (Senin et al., 2006). Furthermore, a transgenic rat line harboring the S344Ter mutation leads a truncated C-terminus of RHO. Since the C-terminus of RHO has multiple vital phosphorylation sites, this model exhibits drastically reduced phosphorylation levels overall, as well as extensive retinal degeneration (Senin et al., 2006).

Rhodopsin is palmitoylated at multiple sites, including Cys322 and Cys323; the research findings of Olausson et al. (2012) suggest that these two sites differentially modify the biochemical properties of RHO, including altering protein binding and flexibility. Although their study mainly comprised simulations, they confirm some of these molecular properties of RHO. Seno and Hayashi demonstrated the significance of RHO palmitoylation, as they discovered that this PTM is a prerequisite for RHO oligomerization (Figure 1B); they also discovered its tendency to incorporate into lipid rafts (Seno and Hayashi, 2017). Importantly, the homodimerization and higher-order oligomerization of RHO could be vital for the dim-light response (Gunkel et al., 2015). Additionally, Wang et al. report that palmitoylation-deficient RHO has altered photodynamic properties, including reduced sensitivity to light flashes and quicker inactivation time (Wang et al., 2005). Sachs et al. (2000) report, palmitoylation may also be critical for RHO activation by all-trans-retinal. Given their evidence asserting the absolute requirement of certain RHO PTMs in visual response, these studies point to just how important PTMs can be. These studies’ research foci represent only a small fraction of the RHO PTMs that have been studied.

Additional PTMs reportedly play a role in RP progression. Like research on glaucoma and DR, work on PTMs in RP have been mainly limited to single proteins rather than large-scale proteomic datasets. Since PRs are so lipid-rich, the lipidation PTMs of key PR proteins has been characterized, but the precise extent to which these PTMs contribute to RP or general retinal homeostasis is not known. For example, the P2Y mutation of progressive rod–cone degeneration (PRCD) interferes with its palmitoylation, drastically alters PRCD stability, and is directly linked to RP (Murphy and Kolandaivelu, 2016). RP 2 (RP2) is a protein, albeit ubiquitously expressed, that is directly implicated in RD (Demers et al., 2015). Demers et al. report that two N-terminal sites of lipidation are vital for RP2’s membrane localization, and this finding aligns with that of a previous study reporting that mutations affecting lipidation (G2V, C3S, and ΔS6) at the N-terminal amino acids not only result in X-linked RP, but also lead to the mistrafficking of these proteins (Chapple et al., 2000). Interestingly, Kumeta et al. report that the ΔS6 mutation not only results in poor membrane localization but also leads to the failure to reach the connecting cilium of PRs (Kumeta et al., 2018). Additional work characterizes mutations leading to RP due to mechanisms that result in the aberrant phosphorylation of key proteins. For example, various mutations in the gene encoding neural retina leucine zipper (*NRL*) leading to a phosphorylation-deficient transcription factor have been identified in patients with ADRP (Kanda et al., 2007).

Studies continue to identify novel genes associated with RP, and some mutations identified in the future will undoubtedly result in PTM changes. The examples discussed here provide a snapshot of the mutations that lead to RP and are associated with PTM changes. Another point is that gene-independent treatments may also be generated for use in RP treatment, and they may work by promoting or inhibiting certain PTMs (similar to the approaches mentioned in the glaucoma section). For example, using gene therapy to overexpress constitutively active or dominant negative mutants may grant the field a method of promoting PR neuroprotection that is independent of the disease-causing mutation. Therefore, understanding how the retina dynamically alters PTMs in response to disease is critical to the development of future therapeutic strategies.

## Conclusion

Phosphorylation is perhaps the most commonly described posttranslational modification (PTM) in the literature pertaining to glaucoma, diabetic retinopathy (DR), and retinitis pigmentosa (RP). Phosphorylation is important to the activation and deactivation of enzymes and receptors, and it can be generally detected through various approaches [e.g., Western blotting with anti-Phosphor–Ser/Thr antibodies, a kinase activity assay, an enzyme-linked immunosorbent assay, intracellular flow cytometry, immunocytochemistry, and mass spectrometry (MS)]. The use of Phosphor–Ser/Thr antibodies against the phosphorylation of individual proteins could significantly help researchers validate multi-analyte kinome profiling obtained through MS.

A further search of the literature identifies a gap in the field investigating the acetylation and methylation of PTM patterns of the diseased retinal proteome. Primarily, these PTM types have been studied for histone proteins, suggesting that in the future, researchers should work to determine the PTM of individual proteins as well as the roles of acetylation and methylation PTMs in protein functions. For example, acetylation neutralizes the positive charge of lysine. This PTM may affect a variety of protein functions, including protein stability, enzymatic activity, subcellular localization, and interactions with other macromolecules in the cell. Therefore, it is critical to acquire knowledge into how the acetylation of single proteins changes their function during retinal pathogenesis.

Less is known about methylation detected by way of methylation-specific antibodies, the mapping of PTM *via* mass spectrometry (MS) or by MS in conjunction with liquid chromatography, and radioactive labeling. Besides controlling the transcriptional program *via* the methylation of histones, the methylation of individual retinal proteins in the diseased retina is not well known. This represents another rich avenue of future research.

Although studies have been undertaken on the glycosylation of retinal proteins, the results of a literature search indicate no profile of modified glycosylated proteins reported for glaucoma, DR, or RP. Such studies are needed to fill the gaps in our knowledge on retinal metabolism and the pathobiology of retinal diseases. In particular, the role of *O*-GlcNAcylation in the diabetic retina should be validated, since recent studies report enhanced glycosylation in DR (Dierschke et al., 2019). In addition, enhanced palmitoylation—which is inherently challenging to detect due to the dynamic processes therein—needs to be investigated in diabetic retinas in detail.

## Summary

Recent advances in proteome studies have made it possible to identify retinal proteomes associated with glaucoma, DR, and RP. Despite breakthroughs in retinal proteomics, developments in the study of the major posttranslationally modified proteins (PTMomes) of the diseased retina remain nascent. Our literature search identified the need to conduct future research on PTMomes and expedite the development of fields that will generate the knowledge, currently missing, that is needed to design therapeutic treatments.

## Author contributions

CS and MG prepared the draft and wrote the manuscript. Both authors contributed to the article and approved the submitted version.
